# Adaptation and Implementation of the Global Scales for Early Development (GSED) Tool in Ethiopia

**DOI:** 10.3390/children12030299

**Published:** 2025-02-27

**Authors:** Kalkidan Yibeltal, Atsede Teklehaimanot, Firehiwot Workneh, Nebiyou Fasil, Sarah K. G. Jensen, Theresa I. Chin, Krysten North, Betelhem Haymanot, Alemayehu Worku, Anne CC Lee, Yemane Berhane

**Affiliations:** 1 Department of Reproductive Health and Population, Addis Continental Institute of Public Health, Addis Ababa 1000, Ethiopia; 2 Tikur Anbessa Specialized Hospital, Addis Ababa University College of Health Science, Addis Ababa 1000, Ethiopia; atsede.teklehaimanot@aau.edu.et; 3 Department of Epidemiology and Biostatistics, Addis Continental Institute of Public Health, Addis Ababa 1000, Ethiopia; firehiwotworkneh@addiscontinental.edu.et (F.W.); yemaneberhane@addiscontinental.edu.et (Y.B.); 4 Department of Global Health and Health Policy, Addis Continental Institute of Public Health, Addis Ababa 1000, Ethiopia; 5 Department of Pediatrics, Harvard Medical School, Boston, MA 02115, USAknorth1@bwh.harvard.edu (K.N.); 6 Division of Developmental Medicie, Boston Children’s Hospital, Boston, MA 02215, USA; 7 Department of Pediatrics, Warren Alpert Medical School of Brown University, Providence, RI 02903, USA; theresa_chin@brown.edu (T.I.C.);; 8 Department of Pediatrics, Global Advancement of Infants and Mothers (AIM) Lab, Brigham and Women’s Hospital, Boston, MA 02115, USA; 9 Addis Continental Institute of Public Health, Bahir Dar 6000, Ethiopia; betelhemhaymanot@addiscontinental.edu.et

**Keywords:** adaptation, Bahir Dar, child development, global, GSED, Ethiopia

## Abstract

Background: The World Health Organization (WHO) recently developed the Global Scales for Early Development (GSED) tool to address the lack of a population-level metric for early childhood development globally. This paper describes learning from the first experience with the translation, adaptation, and implementation of the GSED tool in Ethiopia. Methods: WHO guidelines were followed to translate and adapt the GSED tool to Amharic. Two Ethiopian child health experts were trained as GSED Master Trainers. The tool was forward translated by two independent translators with previous experience in child development assessment, consensus was obtained, and the back translation was reviewed/approved by the WHO. The GSED app was programmed in Amharic and piloted for 40 children aged 6–36 months, followed by its implementation by trained study nurses in a child development study in Bahir Dar. Results: Minor adaptations were made to terminologies and certain items were rephrased in the short and long forms. Modifications were made to physical objects used in the long form to make the items culturally appropriate and familiar. Local examples were used where necessary. The tool was administered to 364 children aged 6–36 months with an average administration time of 50–60 min. Quality control assessments by master trainers showed high agreement with assessments by trained study nurses throughout the study (average agreement: 91%). This study demonstrated the high acceptability and feasibility of the GSED tools. Conclusions: Local adaptations were required to contextualize the GSED tool for the Ethiopian setting. The preliminary experience with the GSED tool in Ethiopia is positive, with high-competency trained staff and ease of administration.

## 1. Introduction

Early childhood development (ECD) covers a period from conception up to school entry and is a time of continuous major brain development and critical acquisition of language, cognitive, motor, and social–emotional skills [1]. Every child should survive, thrive, and develop to his or her full potential, particularly during the first five years of life, which is essential for lifelong health and well-being [2]. Optimal ECD has lifetime beneficial consequences for educational achievement, adult productivity, and population health. Improved early neurodevelopment, therefore, can spark an intergenerational cascade of improved family and child health outcomes [3]. Despite this, every year, 43% of all children under 5 years in low- and middle-income countries (LMICs) (~250 million children) may not reach their developmental potential [4].

Several factors influence early child development, including socioeconomic factors, nutrition, illness, infectious disease, family mental health, the home environment, and the school environment [5,6,7]. However, in LMICs, the biological and psychosocial risk factors accompanying poverty hinder millions of children in these settings from achieving their milestones, thus threatening educational attainment and adult productivity [2]. It is critical to characterize early child development at the population level and understand what factors may optimize the potential of children across different contexts worldwide.

A wide range of child development assessment tools are available; however, they have faced limitations for global application [8,9,10,11]. ECD tools are designed to evaluate various domains of a child’s development, including cognitive, language, motor, and social–emotional skills [9]. Child development assessment tests tend to be bound by cultural contexts, social norms, and language, requiring careful adaptation to the local context [9]. Existing tools are often resource-intensive and inaccessible due to high costs. In order to track ECD at a global level, there is a need for a standardized, adaptable tool to measure early child development at the population level to provide information on how children are developing across diverse settings and geographies.

To address the lack of a population or programmatic measure or metric of ECD, the World Health Organization (WHO) assembled an interdisciplinary and multi-country team to develop the Global Scales for Early Development (GSED), an early childhood development assessment tool, to be used across diverse cultures [12]. The GSED measures of ECD for children up to 36 months of age provide a metric for child development (the developmental score) at both the population and programmatic level, as well as a system for interpreting scores [13]. The WHO’s GSED provides a standardized way to assess early childhood development for children under three years old globally, focusing on developmental milestones across cognitive, motor, socio-emotional, and language domains.

The government of Ethiopia has long acknowledged the importance and value of investing in ECD, since 2005, and developed the first Early Childhood Care and Education Policy Framework in 2010. The national ECD strategic plan is implemented under the child health program, with the major objective that all children grow and thrive in a secured, safe, and nurturing environment [1,14]. In Ethiopia, 60% of children are estimated to be at risk of suboptimal development due to multiple factors, starting with pregnancy, undernutrition, and lack of responsive child care, stimulation, and safety [7]. Thus, there is a need for accurate, culturally appropriate measures of child neurodevelopmental progression in the Ethiopian context to support policies and programs designed to enhance early childhood well-being and address developmental delays at a population level. We introduced the WHO GSED into Ethiopia for the first time and translated and adapted the tool in collaboration with the WHO. This article presents the first-time use of the GSED in Ethiopia, capturing insights into the operational, cultural, and technical aspects of its deployment. By highlighting our experiences, we aim to provide a practical roadmap for future applications in similar settings, ultimately supporting Ethiopia’s efforts to address developmental needs in early childhood and meet the United Nations’ Sustainable Development Goals (SDGs) for child development.

## 2. Materials and Methods

### 2.1. Study Design and Settings

The GSED was adapted for use in the Bahir Dar Child Development (BCD) study, a cross-sectional study that aimed to characterize typical healthy neurodevelopment in the first 5 years of life in Amhara, Ethiopia. In this study, the GSED was implemented in two health centers in urban Bahir Dar, the regional capital, among children aged 6–36 months. According to the Ethiopian Ministry of Health, urban health centers are expected to provide services to a catchment population of 40,000. These health centers provide preventive, curative, and health promotion services through the health care providers stationed at the health centers and through urban health extension workers, who conduct home visits and community outreach services. The GSED adaptation process was led by the Addis Continental Institute of Public Health, a well-renowned public health training and research center, in collaboration with Harvard Medical School, Brigham and Women’s Hospital, and the World Health Organization.

### 2.2. The Global Scales for Early Development (GSED) Measures

The GSED is a multidomain child development assessment measure for children up to 36 months of age for population-level and programmatic evaluation. The tool was designed by the WHO in collaboration with a global network of researchers in ECD, after psychometric modeling of large-scale datasets by assessing 2211 items from 18 validated ECD measures from 32 countries (of which 30 are low- or middle-income; *n* = 66,075 children) [12,13]. The top-performing items were chosen, extensively field tested, and assessed for reliability and validity. The final GSED tools were then validated in a 3-country study; Bangladesh, Pakistan, and the United Republic of Tanzania. The inter- and intra-rater reliability and intraclass correlation coefficients were high (>0.98 for all forms). The GSED forms also correlated highly with Bayley III (r > 0.88 for all domains). Additionally, the age-adjusted GSED correlated positively with HAZ, gestational age, and home stimulation [15,16]. Currently, additional implementation data are being collected in four countries (Brazil, Côte d’Ivoire, the Republic of China, and the Netherlands) to address broader global validity and inform potential revisions of the tool [12,13,17]. The GSED measures were designed to be neutral and valid for any country and culture; however, minor adaptations may be required for specific items in different cultures [18].

The GSED measures consist of a caregiver-reported short form (SF) and a directly administered long form (LF). The GSED uses a standardized metric, the developmental score (D-score), a scale with interval properties, to measure children’s holistic development [18,19]. The SF is recommended for population-level monitoring purposes, while a combination of the GSED SF and LF may be used to increase measurement precision to capture development at the individual level. We adapted and used both the GSED SF and LF. The GSED SF includes 139 items, representing emerging skills and behaviors within the cognitive, motor, language, and social–emotional domains. All items are presented as questions to the caregiver, with binary response options (Yes/No and ‘Don’t Know’) that use start rules based on the child’s age and stop rules based on age and performance. Assessors record the caregiver’s responses, regardless of the assessor’s observations. The GSED LF includes 155 items capturing similar domains to the SF but observed by the assessor, following start and stop rules based on the child’s age and responses. LF items must be observed incidentally, by eliciting the behavior, or both, depending on the item. Items are organized into three grids (A, B, and C) that enable assessors to measure the child’s performance on similar tasks in succession, making the administration easier for both assessors and children. The GSED LF uses a locally constructed and low-cost kit with basic materials that the child interacts with to demonstrate their abilities. The kit is created by local teams with detailed guidance from the WHO. The responses of all LF items are binary (skill observed/not observed). The measures are created to be paper-based and tablet application-based (GSED app, which uses the Open Data Kit), with built-in administration rules and supporting media files.

The adaptation process followed the steps outlined in the GSED v1.0 adaptation and translation guide, consisting of translation, back translation, and thorough discussion of discrepancies with technical experts [18].

## 3. Results

### 3.1. Adaptation Process

**Figure 1:**
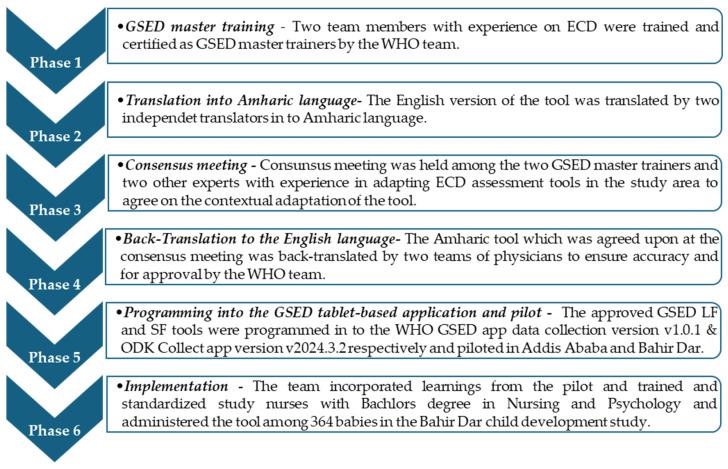
Summary of the adaptation and implementation processes for the GSED tool.

The GSED adaptation and implementation teachings are discussed in six phases, detailed as follows (Figure 1).

#### 3.1.1. Phase 1: GSED Master Training

Two team members participated in the GSED master training, a virtual training organized by the WHO team, and were certified as the first master trainers in Ethiopia. These certified trainers were a pediatrician with expertise in developmental and behavioral pediatrics and ECD (AT) and a physician–researcher with child neurodevelopment expertise (KY). The master trainers were responsible for leading the consensus meeting and the translation and adaptation of the tool into the Amharic language, the official working language of the study area and Ethiopia, as well as for the training of local field teams. Additionally, they were also responsible for leading the pilot testing and implementation of the tool.

#### 3.1.2. Phase 2: Translation into Amharic Language

Permission was obtained from the WHO for the translation of the GSED LF and SF into Amharic and their adaptation to fit the local context. Accordingly, the tools were translated into Amharic by two independent translators concurrently.

The translators were fluent in both the English and Amharic languages. The first translator was an academician and researcher with expertise in psychology and had extensive experience in ECD research, including tool translation and adaptation. This expert was recruited as he was living in the study area and was well acquainted with the culture. The second translator was a certified GSED master trainer.

#### 3.1.3. Phase 3: Consensus Meeting Held with Experts

Following the completion of the tool translation, a consensus meeting was conducted. The meeting consisted of the certified local master trainers and two PhD fellows in applied social psychology and early childhood care and education with prior experience in translating, adapting, and implementing child neurodevelopment assessment tools, including Bayley Scales of Infant and Toddler Development [8], Wechsler Preschool and Primary Scale of Intelligence [18], and International Development and Early Learning Assessment for child development studies in the Amhara region. After reviewing the translated tools individually, the team held a 3-day in-person consensus meeting at ACIPH to ensure that each of the items retained their original meaning after translation and were understandable and culturally appropriate. Based on the discussions conducted during the meeting, an agreement was reached on each item, and the two translations were merged to create one Amharic version of the tool. The numbering system was kept in the original English labeling to ensure consistency with the international versions of the tool and to avoid confusion among assessors.

#### 3.1.4. Phase 3a: Adaptation of GSED Tool for Local Context

During the consensus meeting, the adaptation of certain objects and item terminology was discussed and agreed upon considering the local study context. Below, we have outlined the modifications made to the SF and LF separately.

- Long-Form Adaptations

The long form (LF) is a directly observed assessment, and certain adaptations were required to make the tool culturally relevant for Ethiopia. Below are examples of how the LF items were adapted.

1. Modification of Physical Objects: For item A19, the grasping of small objects with three or four fingers, the local market raisins used were sticky, leading to falsely overrated scores, and local chickpeas were used to replace the items, similar to those used in Bayley Scales of Infant and Toddler Development. The comb and shoe (for items B18/19/30/33/36; requiring identifying objects) were also adapted to locally available items. Procuring two large cups and two small cups with one pair with similar colors and handles was challenging, and thus, our team used cups without handles, which fulfilled the color and size requirements (for item C49), and a separate cup with a handle for activities that required a cup with a handle (for items C11/22). Correctly sized wooden blocks were not possible to procure locally and were brought from abroad. Similarly, our team was unable to obtain shape and peg boards, and these needed to be produced by a carpenter. The LF kit adaptations are summarized in Table 1.

2. Modifications of Cultural and Linguistic Terminology: Several key adaptations were made during the translation and testing phases to ensure the tool’s relevance and understanding in the Ethiopian context. The main modifications made to both the short form (SF) and long form (LF) of the GSED tool are as follows:

Terminology Adjustments:

Caregiver vs. Mother: The Amharic translation for the term “caregiver” was interchangeably used with “mother” with more preference for the term mother to align with local caregiving practices, as mothers usually are the primary caregivers.

Cultural Relevance of Phrases: Certain phrases and developmental concepts were modified to ensure cultural relevance by modifying local examples, e.g., rattle did not have an exact direct Amharic word; thus, we used an Amharic word that was more appropriate for the study area, ‘keshkesh’, based on the sound it produces.

In the study area, the term “water bottle” is not commonly used, but rather water is referred to by specific water brands available in the community. Thus, the team decided to provide credit to the child for naming objects with the use of these commonly accepted water brand names (i.e., ‘highland’, or ‘koda’ in the Amharic version) (item B30).

Local Examples Added:

We also evaluated the appropriateness of examples in the local language and the cultural relevance. The adaptations made to the long form are described in Table 2.

We thoroughly examined the local cultural appropriateness of the visual picture illustrations on the long-form items (items B17, B23, B24, B27, B29, B34, B37, B38, B39, B40, B41, B42, B44, B47, B48, B49), and they were found to be appropriate for the study setting.

- Short-Form Adaptations

The short form (SF) of the GSED includes caregiver reports of child development milestones, and some items require specific cultural and contextual modifications.

Local Examples and Modifications Specific to the Short Form (SF):

Cultural Relevance of Phrases and Terminology Adjustment: Some questions were clearer in English but not in direct Amharic translation. We also considered the local language in the specific study area, the cultural relevance, and the appropriateness of examples in the local language. Based on that, we changed some of the examples for some items. Table 3 shows examples of the adaptations made to the SF.

Pronouns: Since the SF will be administered to the caregiver, we used a unisex pronoun that reflects respect for elders and covers both he/she while referring to the caregiver’s child. For example, rather than saying “lijish” or “Lijih”, we say for both female and male caregivers “lijiwo”.

The adaptations made to the GSED SF are summarized in Table 3.

These linguistic and cultural adaptations played a crucial role in making the GSED tool meaningful in the Ethiopian context. The process highlighted the importance of involving local experts and caregivers in the translation and adaptation process to ensure cultural and linguistic relevance; their insights helped refine the tool to ensure it was both valid and practical for local use. Adjusting examples and terminology to reflect the local culture and experiences of Ethiopian children was crucial. The use of local food examples, common chores, and culturally relevant phrases improved the local understanding of the tool.

#### 3.1.5. Phase 4: Back Translation to the English Language

To ensure consistency, the tool was translated back to English. For the back translation, we recruited four Ethiopian Masters of Public Health fellow physicians at ACIPH. We paired the four translators in two groups and divided the SF and LF proportionally to have two sets of back translations. After completion, the back translation was merged to form one tool with both the LF and SF. Then, the Amharic translation and the back translations were shared in Excel form for a review by the WHO team to ensure the alignment of the Amharic version with the original English version. Feedback was provided by the WHO team on some of the back-translated items for both the SF and LF, where revisions were made to the specific items. The translated GSED SF and LF were approved by the WHO on 21 August and 5 October 2023, respectively.

#### 3.1.6. Phase 5: Programming into the GSED Tablet-Based Application and Pilot Testing

The WHO GSED application provides an easy way of administering the tool, including the start and stop rules, and simplifies the integration of the audiovisual aids [16,19]. The WHO GSED app data collection version v1.0.1 was adapted and used for the GSED LF, whereas the ODK Collect app version v2024.3.2 was used for the SF. The translated tools (both GSED SF and LF) were pilot-tested with caregivers and children in Addis Ababa and Bahir Dar with 40 mother–baby pairs before the implementation. The tool translation was refined following the pilot, mainly to use specific terms that are used in the study context.

#### 3.1.7. Phase 6: Implementation

##### Phase 6.1. GSED Training for Field Study Nurses

We trained six study nurses with nursing and psychology backgrounds on the GSED SF and LF using the WHO training materials aggregated with sample videos and audio recordings, supplemented by mock sessions. The training lasted 15 days and was led by two GSED certified trainers. The training focused on the correct administration and scoring of the GSED tool and the usage of the GSED app. The practical sessions and standardization were conducted in a day-care and health center among babies who came for vaccination. Certification involved quizzes, observations of practical sessions, and co-scoring of assessments. An inter-rater reliability test reflecting ≥80% agreement was required to ensure standardized data collection skills. After 15 days of training, the trainees had high agreement with master trainees, with an average agreement score of 91.1%.

To ensure consistency in the tool’s administration among different settings, the WHO recommends administering the SF questions as they appear on the form without making any edits or adding explanations. Accordingly, the study nurses were trained not to provide any further explanation on the questions even if the mother/caregiver did not understand them.

##### Phase 6.2. Field Data Collection

The implementation of the GSED was conducted from 29 April to 29 November 2024 in two BCD study health centers in Bahir Dar. We conducted 364 GSED SF and 355 GSED LF assessments for the 365 participants that consented. The BCD study enrolled healthy children who visited the health centers for clinical services or accompanying their caregivers based on the study inclusion criteria (without congenital birth defects or hypoxic–ischemic encephalopathy, developmental delay, or behavioral disorders according to maternal report and without clinical signs and symptoms of illness based on parental/child report). The average administration time for the GSED LF was 35–40 min, and for the GSED SF, it was 15–20 min. The tool demonstrated high acceptability by study nurses and participants.

To maintain high-quality data collection and ensure the validity of the results, we implemented several quality control measures throughout the study. Nearly 30% of the GSED LF assessments conducted in the BCD study were video-recorded for quality control after parental consent was obtained. Quality control assessments by master trainers had high concordance with assessments by trained study nurses throughout the study. Additionally, master trainers conducted field supervision to monitor study nurses in the field and provided on-site feedback on tool administration.

## 4. Adaptation and Implementation Challenges and Learnings

The GSED tool was designed to be valid for any country and culture and required minor adaptations for the local/Ethiopian context. Adapting and implementing the GSED tool in Ethiopia provided important lessons regarding cultural adaptation, language translation, and practical application in a resource-limited setting. By addressing challenges related to developmental concept adaptation, terminology changes, and environmental considerations, we were able to make the GSED tool more relevant and usable in Ethiopia. The implementation provided a wealth of learning opportunities. Below, we outline some of the specific challenges we faced and the adaptations we made to ensure the tool’s cultural and linguistic relevance. Key challenges and learnings are described below.

Understanding Developmental Concepts in the Local Context: One of the primary challenges we faced was ensuring that the GSED’s developmental concepts aligned with local cultural practices and expectations. For example, tasks in the motor development section, such as balancing on one leg or crawling, required us to account for the fact that children in rural areas might not be encouraged to engage in such activities as frequently as children in urban settings, as they are mostly carried by caregivers and these children have less opportunity to be on the floor to practice crawling. Some babies were not willing to do item A39 since they perceived kneeling as a punishment. This necessitated adjustments in how tasks were presented and assessed, such as conducting assessments in a more natural setting (e.g., on a mat or the caregiver’s lap) rather than a structured environment and engaging caregivers in demonstrating some items.

Socio-Emotional Development: Socio-emotional concepts were particularly challenging, as expressions of emotion or social behaviors may be perceived differently in Ethiopia compared to other cultures. Social behaviors, like making eye contact or engaging with unfamiliar adults, are less encouraged in some rural communities that require the local team to be more observant of culturally appropriate responses. This was also addressed by performing assessments on caregivers’ lap to allow natural socio-emotional behaviors to emerge.

Environmental and Logistical Adaptations: Even though the study had designated study rooms in both health centers, some children did not want to enter the study room due to fear of injection/shots and fear of white coats. In addition to this, study visits were limited to the health centers, which is an unusual setup for children, impacting children’s behavior and their performance. Thus, the study nurses took their time and used different methods to build rapport with the children, especially using objects from the LF kit. In addition, we mostly conducted assessments on a mat or with the child sitting on their mother’s lap rather than at tables or desks. We quickly learned that these settings not only made the children feel more at ease but also allowed for more natural interaction between the child and the caregiver, leading to more accurate assessments of GSED LF items. In particular, infants and toddlers were more comfortable on their mother’s lap or by their mother’s side on the mat.

Challenges in Administration: We learned that it was a challenge administering most B stream (language domain) items as babies were not willing to talk in the presence of an unfamiliar person and a new environment, even though encouraged by parents. Thus, study nurses tried their best to score items through incidental observations, starting from the moment they met the baby. Another challenge was children getting bored and tired in the middle of the evaluation and crying to go home. This was addressed by prioritizing the GSED SF right after consent, followed by the GSED LF and remaining study questionnaires, to complete tasks with the child as soon as they arrived. Feeding breaks were also given when the children looked tired, and refreshments (cookies and soft drinks) were provided for the mothers as well. The study nurses also worked in pairs and with participating mothers when babies fixated on some items and would not move on to the next assessment. Similar items were also administered consecutively based on the guide to minimize this, and objects related to the specific items were only presented to the child for a specific LF item.

The length of administration time made some children bored or anxious, necessitating breaks for calming or feeding. Some mothers/caregivers found the duration of the assessment unacceptable. On certain occasions, toys distracted the children from following instructions. Mothers sometimes reacted to their children’s actions, causing further interruptions. There was a potential social desirability bias when using the short form, as mothers responded positively.

Tablet Use: The GSED app was designed to simplify data collection and scoring. However, there were challenges initially in programming the GSED LF app in Amharic as the app was using an old version of the ODK template, leading to errors in displaying the instructions for the tasks and showing the stop rules, and it took a few weeks to resolve all the issues. However, in anticipation of tablet use challenges related to app and power outages in a rural setting, study nurses were also trained and prepared to administer the paper-based GSED tools.

## 5. Conclusions

This paper highlights our learning in adapting the GSED tools to fit the Ethiopian context and the importance of engaging local experts during the adaptation process to ensure language clarity and cultural relevance. Our experience with adapting and using the GSED tool in Ethiopia was positive, showing that the GSED is a robust tool that can be administered with minor adaptations, high inter-rater reliability, and ease of administration by trained junior health graduates with no prior experience. The learning gained from the process can serve as a roadmap for future implementations of the GSED tool in other low-resource and culturally diverse settings, contributing to global efforts to enhance early childhood development monitoring.

## Figures and Tables

**Table 1:** Examples of adaptations made to the GSED LF kit in Ethiopia.

Object	Original	Adaptation
Raisin/rice puff	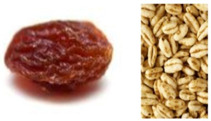	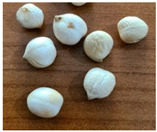
Comb	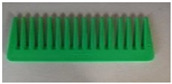	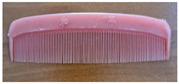
Shoe	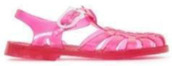	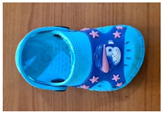
Two large cups: one of each color	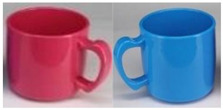	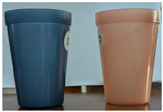
Two small cups: one of each color	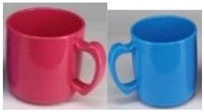	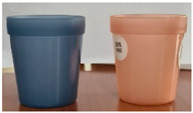
Cup with a handle	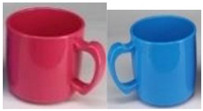	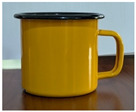

**Table 2:** Global scales for early development long-form adaptations, Ethiopia.

Original Items	Adaptation
Item A13: Use small safe food items (e.g., RICE PUFF/RAISIN/CHEERIO),	Examples were modified to ‘seeds of rice, corn, beans or raisin’
Item B20: Say, “Where’s your/my/caregiver’s ___?”, or “Can you point to your/my/caregiver’s ____?”. You may ask about: (a) shoes; (b) shirt; (c) pants; (d) scarf; (e) skirt. [Can use any of these or another item can be substituted for your local context.]	Mother was primarily used in place of caregiver and local examples of common clothings like “netela”, ”kuta”, “headscarf” were suggested to be used. “where is your mom’s or my…?”or “can you point towards your mom or me?” You can ask about (A) Shoe, (B) Shirt, (C) pants, (D) scarf, (E) dress. (you can use these clothes or ones that are more familiar to the area like “netela”, ”kuta”, headscarf and the like)
Item B31: During testing, record any of the child’s sentences that contain at least 3 words. Common examples include, “Daddy go home”, “Give me toy”, “Do it again”, “I want that” and “Me get ball”. [Examples can be provided for the local setting.]	Examples were modified to make it appropriate for translation “mama give water”, “Dad went home”, “I found a ball”
Item C 39: “Look, let’s make some food”. e.g., rice, noodles, cereal, fruit, and you could pretend to cut a block on the plate with your hand.	Instead of the mentioned foods, culturally specific food examples like shiro wot (chickpea stew), dinich wot (potato stew), and misir wot (lentil stew) were used to make the activity more relatable for children in Ethiopia.

**Table 3:** Global scales for early development short-form adaptations, Ethiopia.

Original Items	Adaptation
SF077: “Can your child break off a piece of food and feed it to him/her-self? [Use local examples of food.]”	Replaced generic food examples with locally familiar foods such as injera and bread, which are staples in Ethiopian households. “Can your child break off a piece of Injera or bread and feed it to him/her-self?
SF 82: Can your child greet people either by giving his/her hand or saying “Hello”? [Use local examples of greetings.]	It was modified to make it culturally appropriate. Is your child able to extend his arm or use words to greet a person? (by bowing down)
SF102: “Does your child help out around the house with simple chores, even if he/she doesn’t do them well? [Use local examples of chores.]”	Examples were modified to reflect common Ethiopian chores, such as fetching a spoon, picking up a fallen object, or helping with washing dishes.
SF136: “Can your child talk about things that will happen in the future using correct language (e.g., ‘Tomorrow he will attend school,’ or ‘Next week we will go to the market’)?”	Examples were added (he will go to school tomorrow, he will go to the market next week, I will take a shower tomorrow)

## Data Availability

Data are available upon reasonable request. The final dataset will be available to the BCD investigative team and collaborators. Anonymized study data may be made available upon request to the study PIs with approval and in accordance with Ethiopian and US regulatory guidelines. The data are not publicly available due to being a part of an ongoing study.
